# Matrix disequilibrium in Alzheimer’s disease and conditions that increase Alzheimer’s disease risk

**DOI:** 10.3389/fnins.2023.1188065

**Published:** 2023-05-26

**Authors:** Matthew Amontree, Samantha Deasy, R. Scott Turner, Katherine Conant

**Affiliations:** ^1^Department of Neuroscience, Georgetown University Medical Center, Washington, DC, United States; ^2^Department of Neurology, Georgetown University Medical Center, Washington, DC, United States

**Keywords:** extracellular matrix, perineuronal net (PNNs), Alzheiemer’s disease, chondroitin sulfate proteoglycan (CSPGs), Matrix metalloproteinases (MMPs), major Depressive Disorder (MDD), Aging, APOE

## Abstract

Alzheimer’s Disease (AD) and related dementias are a leading cause of death globally and are predicted to increase in prevalence. Despite this expected increase in the prevalence of AD, we have yet to elucidate the causality of the neurodegeneration observed in AD and we lack effective therapeutics to combat the progressive neuronal loss. Throughout the past 30 years, several non-mutually exclusive hypotheses have arisen to explain the causative pathologies in AD: amyloid cascade, hyper-phosphorylated tau accumulation, cholinergic loss, chronic neuroinflammation, oxidative stress, and mitochondrial and cerebrovascular dysfunction. Published studies in this field have also focused on changes in neuronal extracellular matrix (ECM), which is critical to synaptic formation, function, and stability. Two of the greatest non-modifiable risk factors for development of AD (aside from autosomal dominant familial AD gene mutations) are aging and APOE status, and two of the greatest modifiable risk factors for AD and related dementias are untreated major depressive disorder (MDD) and obesity. Indeed, the risk of developing AD doubles for every 5 years after ≥ 65, and the *APOE4* allele increases AD risk with the greatest risk in homozygous *APOE4* carriers. In this review, we will describe mechanisms by which excess ECM accumulation may contribute to AD pathology and discuss pathological ECM alterations that occur in AD as well as conditions that increase the AD risk. We will discuss the relationship of AD risk factors to chronic central nervous system and peripheral inflammation and detail ECM changes that may follow. In addition, we will discuss recent data our lab has obtained on ECM components and effectors in *APOE4/4* and *APOE3/3* expressing murine brain lysates, as well as human cerebrospinal fluid (CSF) samples from *APOE3* and *APOE4* expressing AD individuals. We will describe the principal molecules that function in ECM turnover as well as abnormalities in these molecular systems that have been observed in AD. Finally, we will communicate therapeutic interventions that have the potential to modulate ECM deposition and turnover *in vivo*.

## 1. Introduction

In this review, we will describe mechanisms by which excess ECM accumulation may contribute to AD pathology and discuss pathological ECM alterations that occur in AD as well as conditions that increase the AD risk. We will discuss the relationship of AD risk factors to chronic central nervous system and peripheral inflammation and detail ECM changes that may follow. In addition, we will discuss recent data our lab has obtained on ECM components and effectors in *APOE4/4* and *APOE3/3* expressing murine brain lysates, as well as human cerebrospinal fluid (CSF) samples from *APOE3* and *APOE4* expressing AD individuals. We will describe the principal molecules that function in ECM turnover as well as abnormalities in these molecular systems that have been observed in AD. Finally, we will communicate therapeutic interventions that have the potential to modulate ECM deposition and turnover *in vivo*.

## 2. Brain ECM

The neuronal ECM is structurally similar to ECM in other organ systems such as the cardiac myocardium. Both cardiac and neuronal ECMs have a framework composed of fibrous proteins such as collagen, fibronectin, laminin, and elastin. Furthermore within the framework, hyaluronic acid (HA) is abundant and serves as a substrate for proteoglycans with glycosaminoglycan (GAG) attachments such as chondroitin sulfate proteoglycans (CSPGs; Ex: versican). Finally, link proteins such as Tenascin-C and HAPLNs associate CSPGs with HA and with each other to impart stability. The major differences between the non-neural ECM and neural ECM are (1) the presence of CNS specific proteoglycans such as brevican and neurocan and (2) the cell types that are important to the formation and maturation of the ECM (3) the relative abundance of the individual ECM components and (4) the sulphation pattern of CSPGs.

The function of ECM in the CNS is also somewhat unique. CSPG components function to inhibit neuronal plasticity through varied mechanisms. These include inhibition of axonal growth and increased firing of select GABAergic neurons that dampen excitatory neurotransmission (Bozzelli et al., 2018). ECM proteins can also promote inflammation through their ability to interact with microglial CD44 and Toll-like Receptors (Jang et al., 2020). In contrast, CSPGs have been shown to possess neuroprotective properties including anti-inflammatory and anti-oxidant effects (Egea et al., 2010). These divergent properties of CSPGs may occur due to varied sulphation states and bioactive fragments of CSPG proteolysis (Jang et al., 2020).

**Figure 1:**
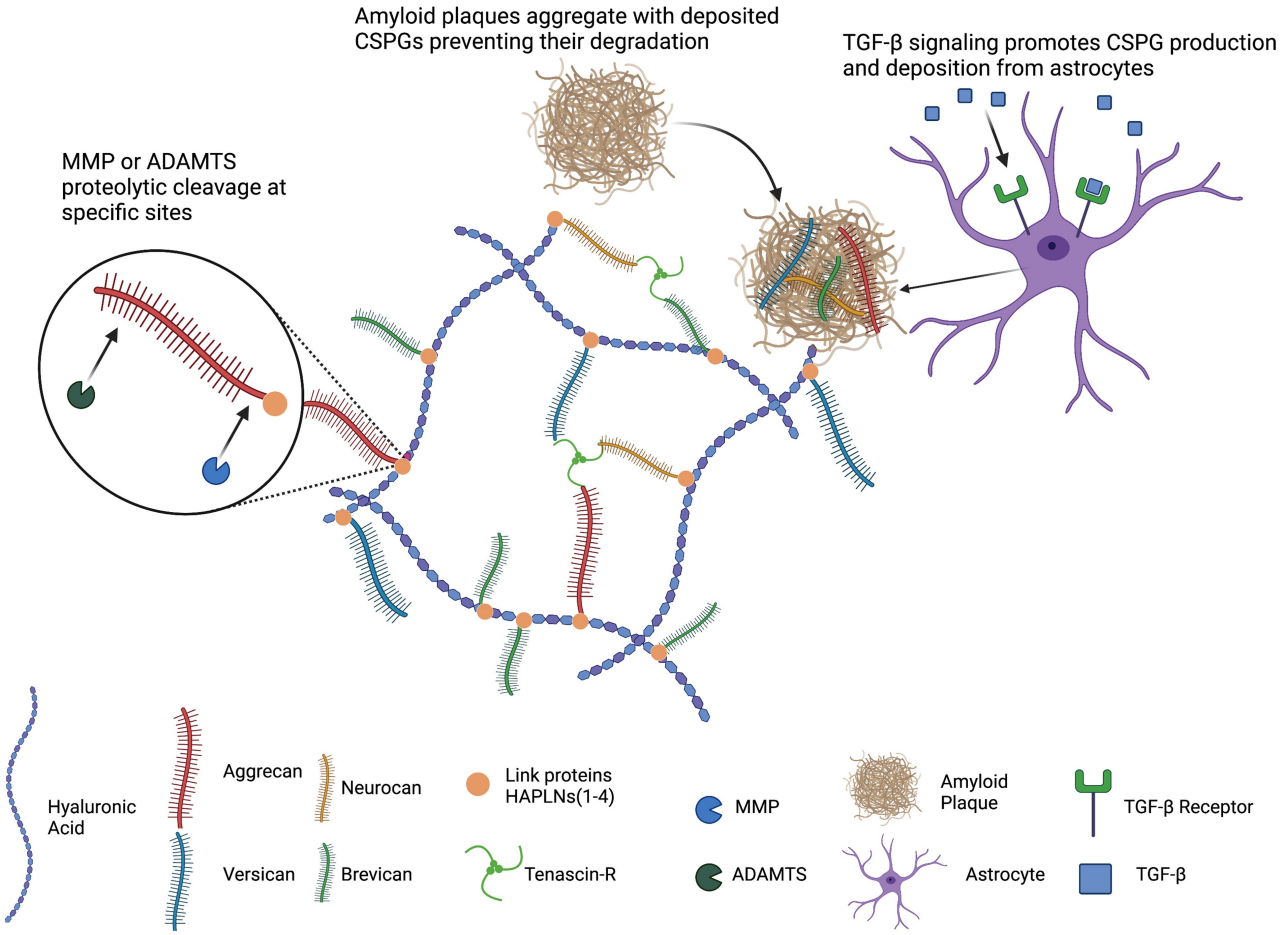
Schematic representation of general PNN structure. Hyaluronic acid (HA) serves as the backbone of PNNs. The lecticans (aggrecan, versican, neurocan, and brevican) associate with HA, link proteins (HAPLNs) stabilize this association, and tenascins cross-links lecticans. TGF-β signaling promotes astrocytic production and secretion of CSPGs. Deposited CSPGs can sequester amyloid plaques and hinder degradation. This image was created using Biorender graphic software (https://Biorender.com).

Perineural Nets (PNNs) are a specialized type of compact brain ECM which predominantly surrounds the soma and proximal dendrites of parvalbumin (PV)-expressing GABAergic interneurons (Bozzelli et al., 2018) (See Figure 1). PNNs generally increase the ability of PV neurons to fire, and thus PNNs can indirectly inhibit the activity of glutamatergic neurons. For example, PNNs have been shown to decrease PV neuron membrane capacitance and increase GluA receptor insertion (Favuzzi et al., 2017; Tewari et al., 2018). PNNs also prevent lateral diffusion of GluA receptors (Frischknecht et al., 2009). Moreover, PNNs may help to localize presynaptic glutamate that is released onto PV expressing cells (Tewari et al., 2023). Consistent with these effects, disruption of PNNs has been linked to an increase in pyramidal cell activity at the single cell and population level (Slaker et al., 2015; Lensjø et al., 2017; Bozzelli et al., 2018; Alaiyed et al., 2020). Removal of PNNs has also been found to increase inhibitory synapses on PV neurons and to inhibit protein tyrosine phosphatase sigma, both of which promote pyramidal disinhibition (Lesnikova et al., 2021; Yang et al., 2021).

## 3. ECM effectors

In the CNS, several systems modulate ECM including matrix metalloproteases and their inhibitors, molecules such as cytokines and chemokines that increase ECM component expression, and sulfatases that influence the susceptibility of PNNs to proteolysis.

The matrix metalloproteinases fall under two major families: matrix metalloproteinases (MMPs) and A Disintegrin and Metalloproteinases *with or without* ThromboSpondin Motifs ADAM(TS). A third family of cysteine proteases termed, Cathepsins (CTs), have also emerged as contributors to ECM homeostasis (Pantazopoulos et al., 2020). Furthermore, serine proteases (uPa, tPa, and plasmin) play a role in direct ECM degradation as well as modulation of the MMP (Tsuji et al., 2005; Zhao et al., 2008) and ADAMTS (Lemarchant et al., 2014) activation cascades.

Endogenous inhibitors of the MMPs and ADAMTSs are tissue inhibitor of metalloproteinases (TIMPs) and alpha-2-macroglobulin (α2M). SERPINs can instead inhibit serine proteases to indirectly reduce levels of active MMPs (Tsuji et al., 2005; Zhao et al., 2008) and ADAMTSs (Lemarchant et al., 2014).

While varied studies have performed *in vitro* digests to examine the susceptibility of ECM components to MMP mediated proteolysis, whether MMPs are expressed in proximity to these components is an unanswered question. However, data from *in vivo* studies with knockouts or specific inhibitors suggests that MMP-9 can attenuate PNN levels in association with a serotonin norepinephrine reuptake inhibitor (Alaiyed et al., 2020) or light reintroduction after visual deprivation/dark exposure (Murase et al., 2017). Of interest, levels of MMP-9 and/or MMP-13 are increased after kainite induced seizures or status epilepticus in which PNN attenuation is also observed (Szklarczyk et al., 2002; Rankin-Gee et al., 2015; Dubey et al., 2017).

ADAMTS proteins have also been implicated in PNN processing. For example, ADAMTS-12 homozygous knock-out (KO) mice show increased neurocan in cortical lysates compared to wild-type (WT) control mice (western blot; Fontanil et al., 2019). In another study of ADAMTS proteins, Demircan et al., found no difference in aggrecan cleavage fragments (~50 and ~60 kDa) amongst the three groups (ADAMTS-4 KO, ADAMTS-5 KO, and WT) following spinal cord injury (SCI) but a decrease in an ~50 kDa brevican fragment was observed at 7 days post-SCI in ADAMTS-4 and ADAMTS-5 KO mice compared to WT. Furthermore, they found a lack of a ~70 kDa versican fragment in both ADAMTS-4 and ADAMTS-5 KO groups which was not observed in the WT group at 7 days post-SCI (Demircan et al., 2014).

Select signaling molecules can also regulate ECM levels and, in particular, promote ECM deposition. These include TGF-β, which is believed to be one of the most potent endogenous regulators of ECM deposition. Following activation and translocation of SMAD transcription factors, both ECM depositing (collagens, fibronectins, TIMPs, PAIs) and degradation proteins (MMPs) are produced with net increased ECM deposition. Consistent with this, in primary rat astrocyte cultures treated with recombinant TGF-β full-length brevican production is increased, and this effect was not seen with treatment of recombinant IL-1β or VEGF (Hamel et al., 2005).

CCL5, which can upregulate TGF-β (Chang et al., 2012), is an additional important regulator of ECM deposition that is linked to fibrosis in varied endorgans (Berres et al., 2010). In a study with a human hepatic stellate cell line, the FDA approved CCR5 antagonist Maraviroc reduced deposition of ECM proteins including fibrillar collagens and it also reduced levels of TGF-β and TIMP-1 (Coppola et al., 2018). Of note, hepatic stellate cells share many characteristics and homeostatic functions with astrocytes including GFAP expression (Schachtrup et al., 2011; Liao et al., 2013).

PNN sulfation also regulates ECM levels. For example, sulfation can significantly impact PNN susceptibility to proteolysis (Foscarin et al., 2017). In the CNS, there are five chondroitin sulfate (CS) sulphation isomers: non-sulfated-CS, two mono-sulphated isomers (4-CS and 6-CS), and two di-sulphated isomers (4,6-CS or 2,6-CS). The mono sulphated chondroitin sulfate proteoglycans (CSPGs) are the major forms in the CNS with a lesser 4/6-CS ratio during development that gradually increases throughout adulthood (Kitagawa et al., 1997). It has been shown that the 4-CS imparts rigidity on the adult CNS and that 6-CS is permissive to axonal growth and plasticity (Lin et al., 2011; Yang et al., 2021) In a recent study, deletion of chondroitin-6 sulfotransferase led to a reduction in 6-CS levels and relatively early-onset age-related memory impairment (Yang et al., 2021).

## 4. Increased ECM deposition has the potential to contribute to AD-associated neurophysiological changes, cognitive impairment, and impaired amyloid clearance

Developmentally regulated deposition of PNNs closes critical periods of neuronal plasticity and tends to stabilize neuronal synapses and networks (Christensen et al., 2021). This stabilizing function may be impaired in CNS disorders where PNNs are disrupted or diminished including schizophrenia (SCZ; Mauney et al., 2013; Enwright et al., 2016), bipolar depression (BPD; Pantazopoulos et al., 2007; Alcaide et al., 2019), and autism spectrum disorder (ASD; Brandenburg and Blatt, 2022). Alternatively, excessive PNN deposition can impair the ability for new learning and may also detrimentally affect neuronal population activity important to learning and memory. With respect to the latter, excess PNN, as determined based on increased fluorescence following WFA immunostaining and an increased percentage of neurons with detectable PNN, has been linked to reductions in the power of gamma oscillations and the abundance of sharp wave ripples, high frequency oscillations that play a role in working memory and memory consolidation (Bozzelli et al., 2018; Alaiyed et al., 2020). In addition, attenuation of PNNs increases gamma power and SWR abundance (Lensjø et al., 2017; Alaiyed et al., 2020), possibly by diminishing PV neuron-mediated GABAergic inhibition of pyramidal cells (Slaker et al., 2015).

Importantly, gamma power is reduced in AD and in animal models of the same (Klein et al., 2016; Murty et al., 2021; Traikapi and Konstantinou, 2021). Gamma power and sharp wave ripple abundance are also reduced in human APOE4-knock-in (KI) mice and the magnitude of gamma power reduction is correlated with the level of subsequent cognitive decline. In a more recent study using wild type or TgF344-AD rats, low and high frequency gamma power were reduced at an early stage in the latter (6 months; low levels of amyloid accumulation; Stoiljkovic et al., 2019; Moradi et al., 2022). PNN and ECM components may also bind amyloid so that its clearance is impaired. For example, an association between CSPGs and plaques have been described in AD brain (DeWitt et al., 1993; Lepelletier et al., 2017; Hebisch et al., 2023). And while ECMs can also sequester and localize beneficial growth factors, recent studies suggest that a variety of profibrotic chemokines including CCL2 and CCL5 are sequestered as well (Hirose et al., 2001; Masuda et al., 2013).

## 5. ECM changes with AD risk factors

### 5.1. ECM changes in aging

ECM changes with age may arise from diverse causes including cell senescence, which occurs in association with chronic fibrotic diseases (Blokland et al., 2020). A senescence-associated-secretory phenotype (SASP) has been described (Sikora et al., 2021), in which secretory products may contribute to direct and indirect ECM pathology (dysregulation of proteases, ECM components, cytokines and chemokines). Indeed, a variety of profibrotic molecules are elevated in association with aging including TGF-β (Tominaga and Suzuki, 2019).

Consistent with this, there are several findings that show alterations in ECM properties with aging. For example, Mafi et al. found an increase in PNN density in aged Norway rats in the inferior colliculus (IC; Mafi et al., 2020), while Brewton et al. found a decrease in PNN density in the auditory cortex of aged mice (Brewton et al., 2016). In aged *Octodon degus* rodents, which can develop AD like pathology, a slight increase in the intensity and the number of both PNN-and PV-positive cells is detected in the entorhinal cortex of those with AD-like pathology compared to aged degus without AD like pathology (Tan et al., 2022). In addition, the degus with AD-like pathology also express increased levels of glial fibrillary acidic protein (GFAP), which is of interest in that activated astrocytes may express increased levels of PNN components.

**Figure 2:**
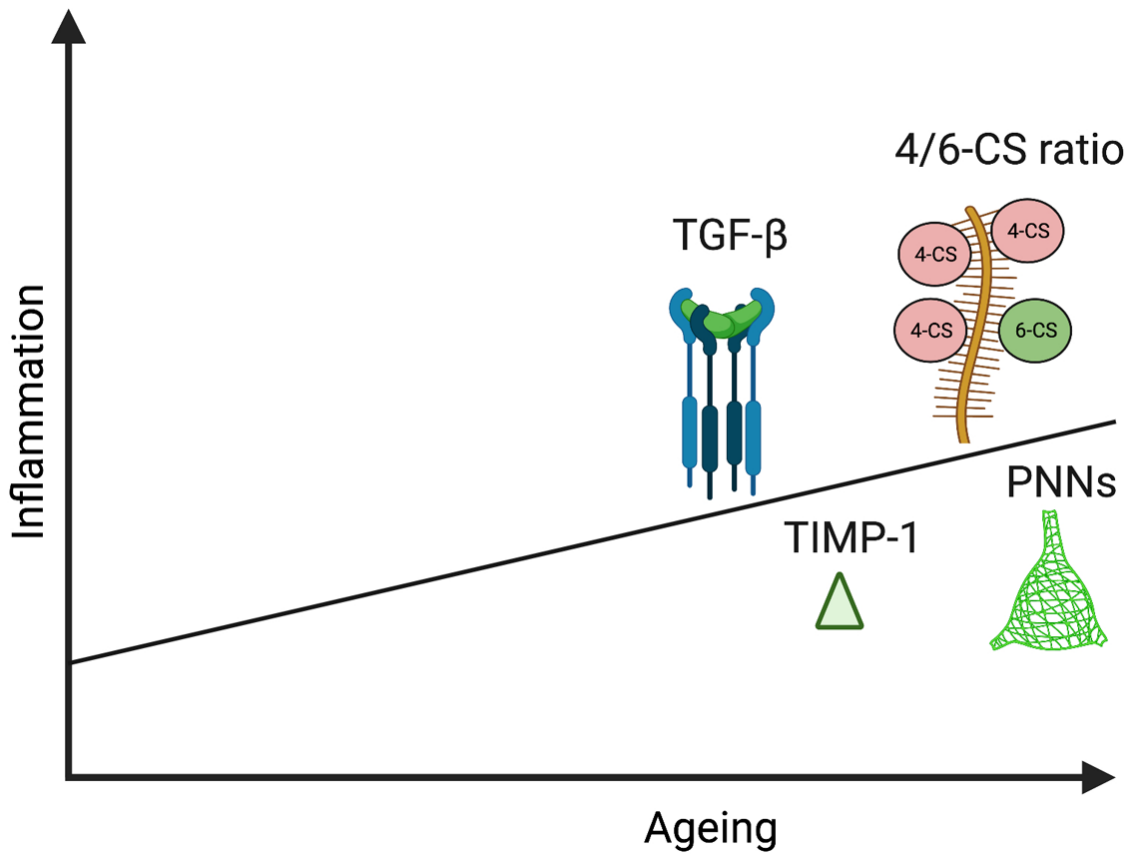
Schematic representation illustrating increase in specific PNN components and effectors with age and inflammation: TGF-β (Fessel, 2019; Yan et al., 2014; Tominaga and Suzuki, 2019), TIMP-1 (Baird et al., 2012; Hasel et al., 2021), 4/6-CS ratio (Foscarin et al., 2017; Baidoe-Ansah et al., 2022), and PNNs (Karetko-Sysa et al., 2014; Végh, Rausell, et al., 2014b; Mafi et al., 2020). This image was created using Biorender graphic software (https://Biorender.com).

As discussed in the section on ECM effectors, there is also an age-related reduction in C6S which renders PNNs more inhibitory (Baidoe-Ansah et al., 2022). This is supported by mechanistic studies in which deletion of chondroitin 6-sulfotransferase simulates aspects of brain aging (Yang et al., 2021). It has been shown that cortical 4-CS increases with ageing in rats and chickens (Kitagawa et al., 1997; Foscarin et al., 2017). Interestingly, in a study of healthy human adults (14–89 years of age), authors noted a significant positive correlation of 4-CS/6-CS ratio with age in synovial joint fluid (Uesaka et al., 2004) and it would be important to determine if this relationship also exists in CSF an overview of ECM effector changes that may occur with aging and inflammation is shown in Figure 2.

### 5.2. ECM changes in APOE4

ECM changes may also occur with APOE expression. The targeted replacement (TR) and KI human APOE mouse models permit us to investigate ECM dynamics independent of Aβ deposition since these mice do not show accumulation of Aβ plaque with age. Astrocytes from these and other mice have been a focus of APOE-directed research as they are the major producers and secretors of brain APOE (Liao et al., 2017). Microglia produce APOE to a significantly lesser extent than astrocytes, though they likely contribute to APOE4-dependent pathology (Shi et al., 2019; Henningfield et al., 2022). Importantly, APOE4 glia produce and secrete less APOE protein than APOE3 and APOE2 glia (Riddell et al., 2008; Lanfranco et al., 2021), which is relevant since APOE is thought to reduce inflammation, a significant driver of overall ECM accumulation (Ulbrich et al., 2021). Our lab has observed increased PNN deposition in retro-splenial cortex and hippocampus of APOE4-TR compared to APOE3-TR (Blanco et al., unpublished observations). Furthermore, we also demonstrated that APOE4-TR mice have decreased ~50 kDa brevican cleavage fragment in CSF samples from human APOE4/4 s compared to APOE3/3 s (Greco et al., 2023). A reduction in this cleavage fragment has also been observed in a murine model of AD (Ajmo et al., 2010).

In other work examining ECM changes with APOE4, Keable et al. found that astrocytes cultured from KI APOE4 mice pups secreted significantly greater amount of fibronectin than KI APOE3 astrocytes and that increased fibronectin could enhance Aβ aggregation in the cerebral microvasculature (Keable et al., 2020). Of interest is that APOE4 murine microglia exhibit significantly increased SERPINA3 mRNA than APOE3 murine microglia that was found in two separate studies (Machlovi et al., 2022; Sepulveda et al., 2022). Serpins can inhibit the activity of ECM degrading proteases. Furthermore, in the same transcriptomic study (Machlovi et al., 2022), they found significantly decreased matrix metalloproteinase-2 (MMP-2) mRNA in APOE4-TR compared to APOE3-TR. Given MMP-2’s role in ECM homeostasis, this evidence supports increased ECM deposition in APOE4 carriers.

Work from our lab has shown increased TIMP-1 and CCL5 in CSF samples from humans expressing *APOE4* and brain lysates from mice with a targeted replacement of this allele (Greco et al., 2023). In addition, recently published work from the Goate lab shows chemokine and matrisome (ECM protein and associated factors) increases in human IPSC-derived APOE4 astrocytes. This work also shows that APOE4 microglia are enriched for ECM, chemokine and cytokine signaling pathways in multiple brain regions (Tcw et al., 2022).

Of additional interest, ECM changes at the level of the blood brain barrier basement membrane may occur with APOE4 (Jackson et al., 2022). The risk of anti-amyloid antibody associated microhemorrhages is increased in APOE4 expressing individuals (Withington and Turner, 2022) and this could be due in part to increased amyloid sequestration by excess basement membrane matrix deposition.

### 5.3. ECM changes in obesity

Obesity is a major risk factor for the development of disorders characterized by excess ECM deposition in endorgans. These include Type II diabetes (T2D) and non-alcoholic fatty liver disease (NAFLD). Furthermore, obesity increases the risk for cognitive impairment, with several studies noting deficits in cognitive domains (working memory, verbal learning, episodic memory) in obese compared to non-obese individuals (Cournot et al., 2006; Gunstad et al., 2006; Fergenbaum et al., 2009; Coppin et al., 2014).

Adverse ECM effects due to obesity may be attributed to metabolic dysfunction, chronic inflammation, and mechanical stress. In T2D ECM pathology may occur in varied endorgans and lead to disorders including diabetic retinopathy, characterized by pathological ECM deposition and basement membrane thickening with progressive blindness (Giblin et al., 2022). In NAFLD, a disorder in which approximately 25% of patients will progress to liver cirrhosis. NAFLD-associated cirrhosis is characterized by intense collagen deposition by activated stellate cells (Fernando et al., 2019), a cell type which shares many common features with CNS astrocytes (Schachtrup et al., 2011). Moreover, in rodent models of NAFLD, activated stellate cells deposit CSPGs such as versican which can inhibit hepatocyte regeneration and recovery from injury (Bukong et al., 2016). Of interest, NAFLD is associated with an increased risk of developing dementia (Filipović et al., 2018; Shang et al., 2022). In addition, advanced liver fibrosis has been associated with increased rhinal Tau(Weinstein et al., 2022). Though the reasons for increased dementia risk in NAFLD are likely multifactorial, with a potential role for impaired cerebral perfusion and hepatic clearance of toxic molecules (Hadjihambi, 2022), it is tempting to speculate that chronic intracerebral inflammation may contribute.

Whether obesity itself is associated excess ECM deposition in the brain warrants further study. The idea is, however, supported by the link between obesity and neuroinflammation, with increased expression of pro-fibrotic molecules such as TGF-β (Yan et al., 2014; Salas-Venegas et al., 2022). It is also supported by studies that have examined effects of obesity in animal models of AD. For example, in a study using a murine model of AD, a high fat diet (HFD) was associated with increases in hippocampal levels of TIMP-1, CCL2, and CCL5 and increased hippocampal astrocyte and microgliosis. Furthermore, these markers correlated with hippocampal TSPO PET signal, a marker of inflammation (Barron et al., 2016). In two separate studies with AD-obesity mouse models, significantly increased cerebrovascular amyloid deposition was also observed (Takeda et al., 2010; Vargas-Soria et al., 2022). Dual AD-obesity mouse models also perform significantly worse on hippocampal-dependent memory tasks (Takeda et al., 2010; Barron et al., 2016). Interestingly, it has also been demonstrated that obese AD mice have increased hippocampal tau hyperphosphorylation compared to obese, AD, and control groups (Platt et al., 2016). Together these studies emphasize potent synergistic effects between obesity and AD pathology that results in amplified amyloid deposition, tau phosphorylation, and neuroinflammation.

### 5.4. ECM changes with stress and depression

While depression is often an initial symptom of AD, long standing recurrent untreated major-depressive disorder (MDD) is thought to be an independent risk factor for AD (Holmquist et al., 2020; Nedelec et al., 2022). While several mechanisms may be at play including corticosterone mediated reductions in hippocampal dendritic arbor (Watanabe et al., 1992; Magarinos and McEwen, 1995; Conrad et al., 1999; Kleen et al., 2006; McLaughlin et al., 2007), it should be noted that recent studies suggest that ECM deposition in increased in the hippocampus with rodent models of the same (Riga et al., 2017; Alaiyed et al., 2020). For example, in a rat model of chronic social defeat–induced persistent stress ECM increases were observed in the hippocampus. In addition, associated cognitive deficits were normalized by chondroitinase ABC (ChABC) injections that attenuated PNN levels (Riga et al., 2017). Moreover, in a murine model of chronic corticosterone induced depression. PNN increases were also observed in the hippocampus and venlafaxine, a serotonin-norepinephrine reuptake inhibitor could normalize PNN levels and excitatory/inhibitory balance (Alaiyed et al., 2020).

In contrast to MDD, BPD is has been associated with reduced PNN levels (Pantazopoulos et al., 2007). Indeed, SSRIs are often contraindicated in BPD as they can precipitate manic episodes (Patel et al., 2015). First line therapy for bipolar disorder (mood stabilizers) differs from that for major depressive disorder, possibly due to differences in the underlying pathology.

Consistent with MDD findings, it is increasingly appreciated that acute stress can upregulate ECM remodeling while chronic stress, a significant risk factor for depression, is instead associated with excess ECM deposition (Ulbrich et al., 2021). ECM deposition and remodeling involves a complex variety of players, as discussed in an earlier section of this review, with players that favor degradation upregulated at early time points following stress or injury and players that shift the balance towards increased ECM deposition expressed as the insult moves to the chronic stage. In support of this, with acute CNS injury, microglia produce a wide array of proteases that function to breakdown ECM components. These include MMP-9, ADAMs and ADAMTSs (Kaushik et al., 2021). Astrocytes instead produce and secrete a variety of ECM components such as collagens, laminins, GAG-linked lecticans such as CSPGs, as well as link proteins and hyaluronan synthase (Ulbrich et al., 2021). Astrocytes also express MMP inhibitors including TIMP-1 (Crocker et al., 2006; Hasel et al., 2021), and this cell type forms the glial scar following traumatic brain injury (TBI; Mira et al., 2021) or stroke (Huang et al., 2014). Thus, a simplified perspective on glia and ECM is that with chronic injury or stress astrocytes serve a “building” role while with acute injury microglia serve a “deconstructing” role in the CNS ECM (Ulbrich et al., 2021).

## 6. ECM and ECM effector changes in AD

There are several findings that show alterations in ECM properties and/or ECM effector levels with AD. ECM changes have been hypothesized to alter Aβ clearance, glial responses to amyloid, and neuronal sensitivity to amyloid (DeWitt et al., 1993; Lepelletier et al., 2017; Hebisch et al., 2023). In autopsy-based studies, CSPGs have been associated with amyloid plaques (DeWitt et al., 1993) and tau tangles. Furthermore, increases in collagen IV, perlecan and fibronectin were found to correlate with amyloid levels (Lepelletier et al., 2017). Palu and Liesi showed increased *α*1 and γ1 laminins in AD frontal cortices that co-localized with reactive astrocytes (Palu and Liesi, 2002). Shimizu et al., observed a 1.6-fold increase in total proteoglycans in the hippocampus and 4.3 fold increase in total proteoglycans in the superior frontal gyrus of AD brains compared to age-matched, healthy controls (Shimizu et al., 2009). In addition, they showed co-localization of proteoglycans with amyloid plaques. Interestingly, in a separate study, a positive correlation between amyloid plaque deposition and ECM components in AD individuals was observed (Damodarasamy et al., 2020).

PNN changes in AD, including altered sulfation patterns are well reviewed, in (Sun et al., 2021; Ali et al., 2022; Fawcett et al., 2022; Logsdon et al., 2022; Scarlett et al., 2022). Logsdon et al., found a significant increase in CS-GAGs in PFC in AD compared to healthy controls (Logsdon et al., 2022). Liddelow’s lab showed increased levels of 4-sulfotransferase, in AD brain-derived astrocytes (Sadick et al., 2022). This likely increases 4S-CSPG levels which are less susceptible than 6S-CSPGs to proteolysis.

While a recent review detailing PNN changes in AD found that a majority of PNN components are upregulated in AD (HA, HSPGs, CSPGs, DSPGs, TNC, and TNR) with two components decreased (reelin and keratin sulfate proteoglycans; Sun et al., 2021), Bruckner and colleagues showed no change in fluorescent intensity using pan-Anti-CSPG antibody in AD frontal and temporal sections compared to controls (*n*_total_ = 12; Brückner et al., 1999). Morawski and colleagues also demonstrated no change in WFA, parvalbumin, brevican, and aggrecan IHC labeling in AD brains (*n* = 12) vs. controls (*n* = 12; Morawski et al., 2012).Differing observations may be explained by the tools used to detect the PNNs. Baig et al. utilized WFA, a lectin stain that binds N-acteylgalactosamine residues of the lecticans (Baig et al., 2005), and Lendvai used an antibody to the protein component of brevican (Lendvai et al., 2013). Furthermore, it is important to recognize that PNN lectican antibodies have epitopes to different parts of the PNN glycoproteins and thus immunohistochemical data may vary across studies due to PNN structural variability coupled with the heterogeneity of antibody epitope recognition sites. Taken together, the observed differences in these human studies may represent an aspect of changing PNN quality and not quantity. Furthermore, this notion of changing quality in AD brains but not quantity of PNNs or PV+ neurons was supported in a recent study of human AD brains where the sulphation code is implicated (Logsdon et al., 2022). Indeed, a recent review proposed an updated model of PNN pathology in AD where it is believed that reduced WFA+ intensity seen in postmortem AD brains is due to structural and sulphation alterations that reduce WFA’s affinity to PNNs; thus, giving the illusion of reduced PNN density (Scarlett et al., 2022). These studies suggest that future investigations examining PNNs in AD humans and rodent models need to address PNN quality changes that cannot be assessed with traditional methods of IHC labeling or Western blot detection.

With respect to ECM changes with amyloid or tau Tg mouse models of AD, two separate studies have found increased hippocampal brevican levels in these models (Ajmo et al., 2010; Végh et al., 2014a). In addition, one group showed a concomitant decrease in the proteolytic-generated ~50 kDa brevican cleavage fragment in hippocampal lysates from Tg mice (APP/Swe) compared to WT controls (Ajmo et al., 2010). It has also been shown that ChABC intrahippocampal injection in AD mouse models alleviates both neuropathology and cognitive deficits (Howell et al., 2015; Yang et al., 2015). In particular, contextual-fear learning and hippocampal slice long term potentiation (LTP) are increased in ChABC treated APP/PS1 versus penicillinase treated controls (Végh et al., 2014b). ChABC also reduced amyloid load and increased synaptic density in the APP/PS1 mice (Howell et al., 2015). In two separate AD-tau mouse models, ChABC treatment also improved cognitive deficits as assessed by object recognition test and increased field excitatory synaptic potential amplitudes in perirhinal cortex (Yang et al., 2015).

In terms of ECM effectors, analysis of CSF samples from sporadic cerebral amyloid angiopathy (CAA), which shares parallels with AD and can be coincident with the same (Vervuurt et al., 2023), shows a significant reduction in the MMP-2/TIMP-2 ratio, a change that would favor ECM deposition. Indeed, individuals who are afflicted with CAA have increased deposition of matrix at the blood brain barrier basement membrane. Previous studies also suggest that MMP-9 levels may be unchanged or reduced in AD and/or *APOE4* patients as compared to controls (Adair et al., 2004; Mroczko et al., 2014). And though other reports show that MMP-9 may be elevated at the blood brain barrier with *APOE4* or aggressive mouse models of AD (Halliday et al., 2016; Weekman and Wilcock, 2016; Montagne et al., 2020), potential confounds include elevated amyloid levels in aggressive murine models and/or select *APOE4* patient populations (Deb and Gottschall, 1996). In a recent study, we did not see changes in MMP-9 with *APOE4* genotype (Greco et al., 2023). Importantly, the ability of MMP-9 to ameliorate or exacerbate disease pathology is likely a function of quantity as well as localization. For example, increased expression of MMP-9 by activated microglia or pericytes at the blood brain barrier could have detrimental effects, while neuronal-derived and localized MMP activity may target preferentially target PNNs and synaptic adhesion molecules to enhance plasticity (Tian et al., 2007; Conant et al., 2015; Martin-de-Saavedra et al., 2022). This is supported by animal studies in which neuronal expression of MMP-9 was associated with an increase in non-amyloidogenic alpha-secretase cleaved amyloid precursor protein and well as plaque reduction and improved cognition in 6 month old female 5xFAD mice (Fragkouli et al., 2014). In contrast, another study showed that a pharmacological inhibitor of MMP-9, which would also target microglial and pericyte-derived enzyme activity, improved cognition but had no effect on plaque load in the 5XFAD model (Ringland et al., 2021).

## 7. Therapeutic manipulation of PNNs

Following the experiments that provided mounting evidence of PNN’s inhibitory role in neuroplasticity, there is clinical interest in targeting PNNs for disruption to promote recovery in neurological pathology. Intracerebral injection of ChABC in aged rodent models improves memory-dependent behaviors in a variety of AD models. Furthermore, in a spinal-cord injury (SCI) model, local ChABC injection improved functional outcome and recovery (Bradbury et al., 2002). However, ChABC is a bacterial enzyme, thermally instable, and requires continuous injections for maintenance of PNN levels. In this review it is beyond our scope to address all avenues of therapeutic PNN manipulation.

Selective serotonin reuptake inhibitor (SSRIs) and selective serotonin-norepinephrine reuptake inhibitors (SNRIs) are typically used for management of anxiety and depressive symptoms; however, a full understanding of therapeutic mechanisms is lacking. Of interest, these medications have been shown to modulation PNN integrity. In accordance with this hypothesis, our lab has previously shown that MMP-9 dependent attenuation of PNNs can impact physiological markers associated with learning and memory deficits. Using a corticosterone-based mouse model of stress, Alaiyed et al. (2020) demonstrated an increase in expression of PSD-95, an increase in expression of MMP-9, increased pyramidal cell arborization, and increased gamma power in male C57BL/6 J mice treated with the antidepressant Venlafaxine (VFX). In VFX treated MMP-9 null mice, these effects were not seen (Alaiyed et al., 2020). These findings support a requirement for MMP-9 in effecting physiological PNN remodeling *in vivo*.

The successful use of antidepressant medications for modulation of PNNs has also been shown with SSRIs including fluoxetine (FLX). In two separate studies (Ohira et al., 2013; Guirado et al., 2014), FLX treated mice showed decreased PNN density and PV+ neurons in both mPFC and in hippocampus compared to vehicle. In concurrence with these findings, Mukhopadhyay et al., 2021 found a decrease in CA1 and CA3 hippocampal PNNs following FLX treatment in Sprague Dawley rats (Mukhopadhyay et al., 2021). Moreover, Ohira et al., 2019 found reduction of DG and CA3 hippocampal PNN density and PV+ interneurons in FLX treated marmosets (Ohira et al., 2019). Through both attenuation of PNNs and consequent effects on PV interneuron excitability, antidepressant medications may be of use in a combination treatment for the maintenance of plasticity as well as the prevention/alleviation of depressive symptoms commonly associated with AD (Dityatev et al., 2010; Donato et al., 2013).

Targeting PNN sulphation has also shown promise in two separate studies. Pearson et al., found that administration of Aryl-Sulfatase B (ARSB), selectively cleaves 4-CS groups on CSPGs, improved neurite outgrowth *in vitro* and regeneration of optic nerve lesion *in vivo* (Pearson et al., 2018). Furthermore, they found that post-fixed mouse brain sections incubated with ChABC drastically decreased PNN density whereas incubation with ASRB did not change PNN density. In a more recent study Yang et al., stereotaxically delivered an AAV-*chst3* (encodes 6-sulfonotransferase) to perirhinal cortex (PRh) of aged C57BL/6 mice, and found recovery in memory impairment (Yang et al., 2021). In addition, they demonstrated reduction in PV+ neurons and PNN density in AAV-chst3 compared to AAV-GFP control group.

4-methylumbelliferone (4-MU) is an HA synthesis inhibitor approved in Europe for treatment of biliary spasms and demonstrates well-tolerance at high doses in humans. 4-MU functions to inhibit hyaluronic acid (HA) synthesis. Dubisova et al., examined its effects on PNNs in healthy adult C57BL/6JOlaHsd mice given 6-months of oral 4-MU in chow (Dubisova et al., 2022). They found a 72% reduction in GAG content in the brain and 50% reduction in spinal cord compared to controls. Furthermore, 4-MU treated mice showed improved hippocampal-dependent memory performance (spontaneous object recognition task and spontaneous alteration test).

In several studies, ketamine has shown ability to modulate PNNs *in vivo*. Matuszko et al., showed a reduction in PNN density and PV expression in mPFC of low-dose ketamine injected male SD rats (Matuszko et al., 2017). In a follow-up study, the same group demonstrated that PNNs were more numerous but immature in structure (less circular and smaller; Kaushik et al., 2021). Venturino et al., demonstrated that a single, high-dose ketamine injection was sufficient to decrease PNN density in C57BL/6 mice and this effect was increased with frequency of ketamine injections (Venturino et al., 2021). The authors attributed this mechanism of PNN reduction to increased microglia phagocytosis and proteolysis of PNNs; moreover, this was supported by lack of PNN reduction with ketamine when microglia were pharmacologically depleted (PLX5622) or inhibited (clopidogrel). In addition, they showed that 60 Hz gamma entrainment (2 h/day of light flickering) for 5 days drastically reduced PNN density and increased neuronal and microglia MMP-9 immunoreactivity proximal to PNNs.

An alternative approach to gamma entrainment, for non-invasive manipulation of PNNs *in vivo*, is repetitive transcranial magnetic stimulation (rTMS). Zheng et al., demonstrated efficacy of this approach in rats in which rTMS decreased cortical PNN density compared to the sham stimulated group (Zheng et al., 2023).

Given the role of inflammatory soluble mediators in driving ECM deposition and remodeling: inhibition of the CCL5/CCR5 signaling axis may show potential in modulating PNNs. This is supported by recent studies showing maraviroc’s (CCR5 antagonist) ability to attenuate liver fibrosis in a murine model of chronic liver failure. *In vitro* treatment of hepatic stellate cells with maraviroc drastically decreased PNN effectors including TIMP-1, TIMP-2, and TGF-β (Coppola et al., 2018). We found decreased hippocampal TIMP-1 in APOE4/CCR5KO heterozygous mice as compared to age-matched APOE4/WT mice, which supports a physiologic role of the CCR5 axis in modulating TIMP-1 levels (Greco et al., 2023). Interestingly it was shown that humans treated with maraviroc or with the CCR5 mutation specific null-allele (delta32) have improved cognitive and functional recovery following stroke compared to non-carriers (Joy et al., 2019). Future studies should address maraviroc’s clinical application in neurodegenerative disorders such as AD where ECM homeostasis is affected.

Potential therapies to target PNN levels are summarized in Table 1.

**Table 1:**
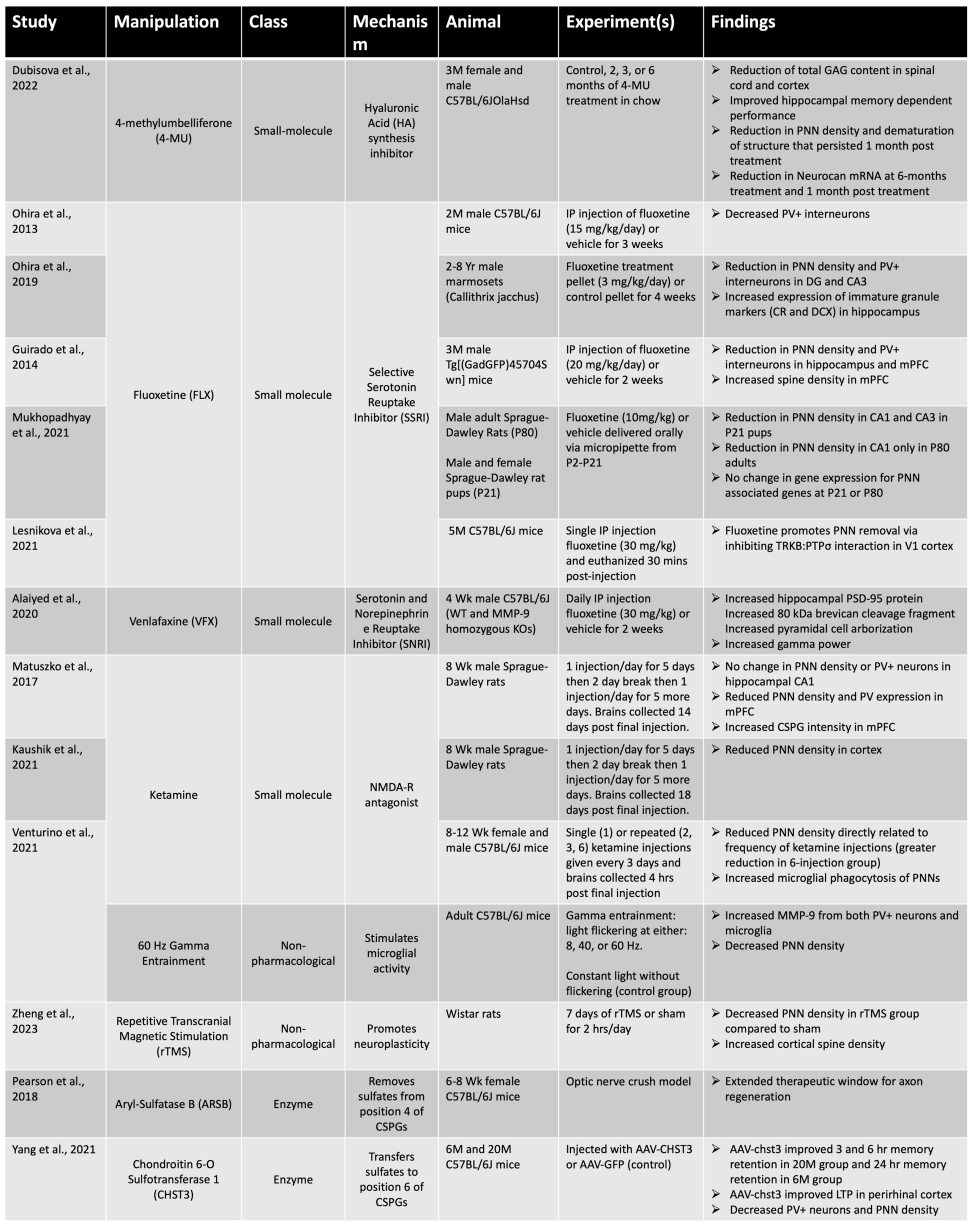
Summary of PNN attenuating strategies for potential clinical use.

## 8. Summary/conclusions/future directions

Numerous epidemiological and genetic studies have implicated a variety of common risk factors for late onset AD. These include age and APOE genotype as well as untreated MDD and obesity.

In this review, we have highlighted chronic inflammation with increased ECM deposition as a shared feature of these predisposing conditions. Since changes in ECM quality and quantity can have adverse physiological effects, we further suggest that excess ECM deposition can restrict neuroplasticity to in turn diminish cognitive reserve. Moreover, increased PNN deposition may alter excitatory/inhibitory balance to impair gamma oscillations and working memory. We have also touched on studies that suggest some ECM proteins may sequester amyloid and thus impair its clearance. Future studies are warranted to test ECM specific interventions in AD and AD risk factor models. Studies could also characterize specific ECM and PNN changes following traumatic brain injury (TBI), which also increases AD risk (Griffiths et al., 2020; Livingston et al., 2020). Of interest, TBI has been associated with chronic inflammation, increased TGF-β, and increased C-4S levels (Bhattacharyya et al., 2015). Future studies are also warranted to determine whether additional conditions associated with chronic brain inflammation, such as long COVID or HIV infection, impair cognition in part through effects on brain ECM.

## Author contributions

MA, SD, and KC conducted the literature search, drafted the manuscript, and prepared the figures. RT provided the human AD CSF samples and assisted in intellectual content. All authors contributed to the article and approved the submitted version.

## Funding

This work was supported by the National Institutes of Health under R01AG077002.

## Conflict of interest

The authors declare that the research was conducted in the absence of any commercial or financial relationships that could be construed as a potential conflict of interest.

## Publisher’s note

All claims expressed in this article are solely those of the authors and do not necessarily represent those of their affiliated organizations, or those of the publisher, the editors and the reviewers. Any product that may be evaluated in this article, or claim that may be made by its manufacturer, is not guaranteed or endorsed by the publisher.
